# One-pot synthesis of ethylmaltol from maltol

**DOI:** 10.3762/bjoc.21.212

**Published:** 2025-12-29

**Authors:** Immanuel Plangger, Marcel Jenny, Gregor Plangger, Thomas Magauer

**Affiliations:** 1 Department of Organic Chemistry and Center for Molecular Biosciences, University of Innsbruck, Innrain 80−82, 6020 Innsbruck, Austria; 2 Red Bull Central Laboratory, Betriebsgebiet Hängender Stein 1, 6713 Ludesch, Austria; 3 Red Bull Service GmbH, Am Brunnen 1, 5330 Fuschl am See, Austria

**Keywords:** ethylmaltol, flavor enhancer, maltol, methylation, 4-pyrones

## Abstract

A novel route to the flavor enhancer ethylmaltol, a synthetic 4-pyrone, from naturally abundant maltol is disclosed. Two strategies were explored for the required C1 homologation. The most direct approach, C–C bond formation via methylation of a dianionic intermediate, proved unsuitable due to competing overalkylation and the necessity of sub-zero temperatures. In contrast, a transient protecting group approach enabled selective methylation under mild conditions. This culminated in a scalable, operationally simple one-pot synthesis of ethylmaltol from a renewable precursor.

## Introduction

**Scheme 1:**
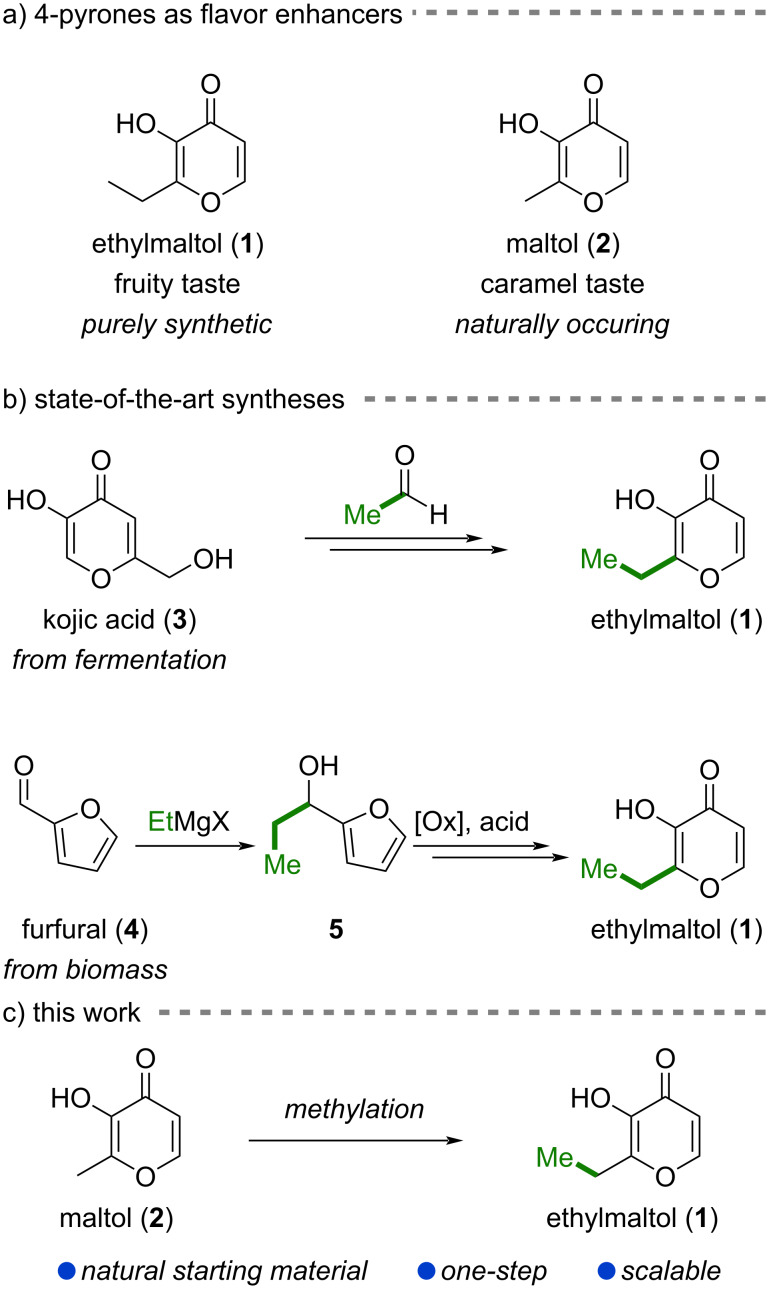
Importance and synthetic approaches to ethylmaltol (**1**). (a) Ethylmaltol (**1**) is widely used as a flavor enhancer. (b) Reported syntheses. (c) One-step synthesis from naturally occurring maltol (**2**) presented in this work.

In 1969, a Pfizer patent first disclosed ethylmaltol (**1**) as a powerful, purely synthetic flavor and aroma enhancer (Scheme 1a) [1]. It has been found to have a 6-times higher flavor-enhancing power compared to its naturally occurring congener maltol (**2**), which has a more caramel-like odor compared to the fruitier ethylmaltol (**1**). Owing to its strong ability to enhance the taste and odor of food, ethylmaltol (**1**) is used as an additive in a wide range of beverages, perfumes, ice cream, cakes, confections, e-liquids, cigarettes, and oral medications. In the European Union, ethylmaltol (**1**) has the food additive code E 637 and due to its lack of toxicity is approved to be dosed *quantum satis*, which means as much as necessary with typical concentrations ranging between 1 to 100 ppm depending on the application [1–3].

Numerous syntheses of ethylmaltol (**1**) have been developed, most of which can be categorized into two distinct approaches based on the respective starting materials (Scheme 1b): In the first case, a 4-pyrone such as kojic acid (**3**), readily available through fermentation, or a synthetic intermediate derived thereof is being utilized. For example, the 1969 Pfizer patent describes the oxidation of kojic acid (**3**) with molecular oxygen to comenic acid, which is then decarboxylated to yield pyromeconic acid. An aldol addition with acetaldehyde followed by reduction of the newly introduced secondary hydroxy group affords ethylmaltol (**1**) in four steps overall [1,4]. Different modifications of this approach have been developed, including performing the aldol addition before decarboxylation [5] or using different feedstock chemicals such as furfuryl alcohol from corncob to access pyromeconic acid [6].

The second group of synthetic strategies takes advantage of furfural (**4**), which originates from the acid-catalyzed dehydration of agricultural biomass. It is then converted with an ethyl Grignard compound to alcohol **5** [7]. From alcohol **5**, oxidation state adjustment through an Achmatowicz rearrangement [8] initiated by either anodic oxidation [7,9–10], chlorine gas [11–12], or *tert*-butyl hypochlorite [13] provides pyran-3-ones. Further oxidation and acid- or heat-induced rearrangement furnishes ethylmaltol (**1**). Of note, some of these procedures allow for one-pot conversion of alcohol **5** to ethylmaltol (**1**) and various patents have focused on this transformation [14–19].

While maltol (**2**) can be obtained through analogous sequences from kojic acid (**3**) [4,20–21] or furfural (**4**) [7,9,11–13], it is naturally occurring in the leaves, needles, and bark of certain trees. In particular, the bark of larch trees contains 0.1–2 wt % maltol (**2**) and economic isolation methods using for example aqueous tannin liquors with extraction efficiencies of >90% have been developed [22–24]. Owing to the natural availability of maltol (**2**), selective methylation of maltol (**2**) would represent the most direct access to ethylmaltol (**1**). Herein, we disclose an operationally simple, one-pot methylation procedure to access ethylmaltol (**1**) from maltol (**2**, Scheme 1c).

## Results and Discussion

Inspired by the selective γ-alkylation of dianions of β-keto esters [25], we initially envisioned the formation of dianion **I** from maltol (**2**), which should undergo selective *C*-methylation with methyl iodide to furnish ethylmaltol (**1**) (Table 1). Typical deprotonation conditions employed for β-keto esters, i.e., sequential treatment of maltol (**2**) with equimolar amounts of sodium hydride and *n*-butyllithium followed by trapping with methyl iodide afforded mostly recovered maltol (**2**, 75%) along with traces of desired ethylmaltol (**1**, Table 1, entry 1). We attributed the low conversion rate to the poor solubility of the in situ-generated sodium maltolate and instead opted for deprotonation with lithium reagents. Attempted double deprotonation with methyl lithium afforded a complex reaction mixture, from which ethylmaltol (**1**) was isolated in 11% together with an inseparable impurity tentatively assigned as **6** based on NMR analysis (Table 1, entry 2). Switching to the less nucleophilic and sterically more hindered base lithium diisopropylamide (LDA), the yield of ethylmaltol (**1**) increased to 25% and maltol (**2**) was recovered in 17% (Table 1, entry 3). Notably, treatment of maltol (**2**) with LDA resulted in a deep purple solution with no apparent solubility issues. We hypothesized that decomposition pathways including potential instability of dianion **I** were responsible for the low overall recovered mass balance. This could be improved by lowering the temperature to −20 °C, which increased the yield of ethylmaltol (**1**) to 46% along with 1% putative **6** and 10% recovered maltol (**2**) (Table 1, entry 4). Reducing the temperature to −30 °C had no significant effect on the reaction outcome (see Supporting Information File 1). Increasing the LDA equivalents even further enabled full conversion, however, in the process significant amounts of overalkylation products such as **7** (8%) and putative **6** (10%) formed (Table 1, entry 5). Both **7** and putative **6** likely arise from dianion interconversions, potentially mediated by LDA, that proceed at rates comparable to the subsequent methylation events. Of note, no *O*-alkylation side products were observed, which we attribute to the lower nucleophilicity of the intermediate lithium alkoxides and to the softer nucleophilic character at C vs O for lithium dienolates. While the dianion strategy was able to convert maltol (**2**) to ethylmaltol (**1**) in yields of up to 57%, the reactions suffered from an unsatisfactory purity profile. First, interconversion between dianionic species led to the formation of, in part, inseparable overalkylation products. Second, excess of base was required for complete conversion of maltol (**2**), which in turn promoted further overalkylation. These challenges, combined with the need for sub-zero temperatures, prompted us to explore a modified and more selective approach with milder reaction parameters.

**Table 1:** Initial investigation of the *C*-methylation of dianions was plagued by competitive interconversion of anionic species and the formation of inseparable overalkylation products.

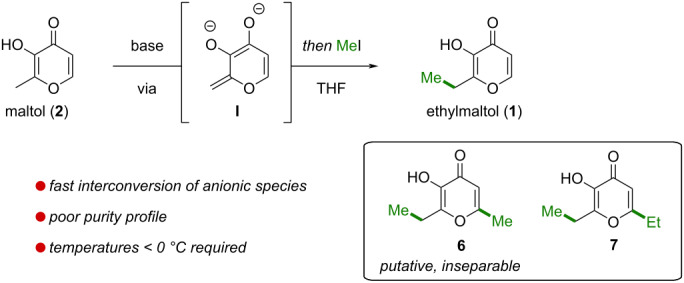					

			yield		
			
entry	base	Temp.	**1** (**6**)^a^	**7**	rec. **2**

1	NaH, then *n*-BuLi	0 °C → 22 °C	3%	n.d.	75%
2	MeLi	−20 °C	11% (2%)	n.d.	2%
3	LDA (2.1 equiv)	0 °C	25%	n.d.	17%
4	**LDA (2.2 equiv)**	**−20 °C**	**46% (1%)**	n.d.	10%
5	**LDA (3.0 equiv)**	**−20 °C**	**57% (10%)**	8%	n.d.

^a^Yield of an inseparable impurity, tentatively identified as **6**, is indicated in brackets. n.d. = not detected.

In our revised strategy, we planned to introduce a transient protecting group for the free hydroxy group, thereby avoiding the necessity of dianion formation with the aim of improving solubility and stability (Table 2). We commenced our investigation with acetylation of maltol (**2**) using acetic anhydride to afford ester **8a**. Unfortunately, the acetate group proved labile upon treatment of **8a** with lithium bis(trimethylsilyl)amide (LiHMDS), resulting exclusively in deprotection to give maltol (**2**, Table 2, entry 1). Turning to the more stable methyl carbonate **8b**, readily obtained from maltol (**2**) via treatment with methyl chloroformate in the presence of base, we found that methylation under typical conditions (LiHMDS, MeI) yielded only a complex product mixture (Table 2, entry 2). Methyl ether **8c** was identified as a suitable substrate for methylation, affording *O*-methyl ethylmaltol (**9c**) in moderate yield (45%, Table 2, entry 3). Subsequent *O*-demethylation of **9c** to ethylmaltol (**1**) was achieved with boron tribromide in dichloromethane in 65% yield. While an *O*-methyl group proved successful, the unsatisfactory overall efficiency to ethylmaltol (**1**) combined with the rather harsh deprotection conditions, led us to investigate other masking groups. Next, we turned towards silyl protecting groups as they offered (a) facile tuning of stability and (b) the opportunity for acid-mediated deprotection during aqueous work-up. Subjecting maltol (**2**) to trimethylsilyl chloride and triethylamine furnished the corresponding silyl ether **8d** in nearly quantitative yield (Table 2, entry 4). Due to the instability of the trimethylsilyl ether functionality to flash column chromatography, methylation of **8d** was followed by desilylation with aqueous hydrochloric acid in a one-pot protocol. To our surprise, this consistently resulted in a 1:1 mixture of ethylmaltol (**1**) and maltol (**2**), each isolated in 30% yield. Assuming insufficient silyl stability during the methylation step, we opted to investigate the bulkier *tert*-butyl(dimethyl)silyl (TBS) group. Protection of maltol (**2**) with *tert*-butyl(dimethyl)silyl chloride (TBSCl) afforded silyl ether **8e** in 99% (Table 2, entry 5). Indeed, methylation of the lithium dienolate of **8e** with methyl iodide at 0 °C afforded TBS-protected ethylmaltol (**9e**) in 64% with no trace of maltol (**2**). Subsequent hydrolysis of **9e** furnished ethylmaltol (**1**) in 95% yield. Screening of different bases for the methylation step of **8e** revealed LiHMDS to be superior to NaHMDS. Various *tert*-butoxide bases as well as 1,8-diazabicyclo[5.4.0]undec-7-ene (DBU) were ineffective for deprotonation (see Supporting Information File 1). Additionally, performing the methylation at 0 °C proved higher yielding than at 22 °C (64% vs 46%).

**Table 2:** Screening of a transient protecting group strategy to access ethylmaltol (**1**).

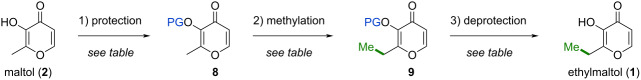				

entry	PG	1) protection	2) methylation	3) deprotection

1	**a**, Ac	Ac_2_O, AcOH 99% **8a**	LiHMDS, then MeI 54% maltol (**2**)^a^	–
2	**b**, CO_2_Me	ClCO_2_Me, NEt_3_ 44% **8b**	LiHMDS, then MeI complex mixture	–
3	**c**, Me	**8c** is commercially available	LiHMDS, then MeI 45% **9c**	BBr_3_ 65% ethylmaltol (**1**)
4	**d**, TMS	TMSCl, NEt_3_ 99% **8d**	LiHMDS, then MeI used crude	aq. HCl 30% ethylmaltol (**1**), 30% maltol (**2**)
5	**e**, TBS	TBSCl, DMAP, NEt_3_ 99% **8e**	LiHMDS, then MeI 64% **9e**	aq. HCl 95% ethylmaltol (**1**)

^a^Yield determined by ^1^H NMR.

**Scheme 2:**
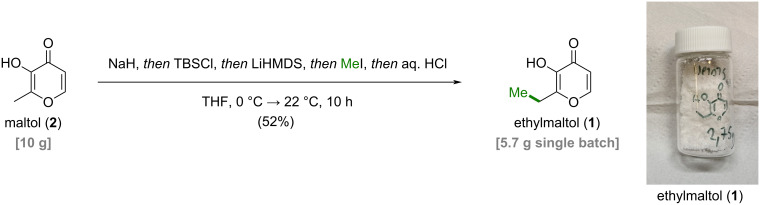
Optimized one-pot procedure to access ethylmaltol (**1**) via a transient protecting group strategy.

Having established a suitable masking group along with optimized methylation conditions, we set out to develop a one-pot protocol for the conversion of maltol (**2**) to ethylmaltol (**1**) (Scheme 2). In our finalized procedure, maltol (**2**) was deprotonated with sodium hydride followed by treatment with TBSCl to in situ obtain silyl ether **8e**, which was subsequently deprotonated with LiHMDS and *C*-methylated with methyl iodide at 0 °C. Addition of aqueous hydrochloric acid enabled silyl deprotection to ethylmaltol (**1**) in 52% yield.

Compared to the previous dianion approach, the developed process produced ethylmaltol (**1**) with a significantly improved purity profile and without the need for sub-zero temperatures. The scalability of this process was showcased by conversion of 10 g maltol (**2**) to 5.7 g ethylmaltol (**1**). Purification with flash column chromatography provided analytically pure ethylmaltol (**1**), which, however, displayed an off-white/yellowish color. A final vacuum distillation yielded purely white ethylmaltol (**1**) consistent with commercial samples. The ethylmaltol (**1**) thus produced was analyzed by the Central Laboratory of Red Bull Service GmbH and was found to meet the quality parameters of commercial ethylmaltol (**1**), posing no concerns from a food safety perspective (see Supporting Information File 1).

## Conclusion

In conclusion, we have developed a new synthetic approach to the flavor enhancer ethylmaltol (**1**) from its naturally occurring congener maltol (**2**). Initial efforts focused on the methylation of a dianionic intermediate, reminiscent of γ-alkylation of β-keto esters. Although this successfully provided ethylmaltol (**1**), the presence of inseparable impurities and the need for industrially unattractive sub-zero temperatures rendered the strategy undesirable. Subsequent exploration of a transient protecting group strategy culminated in a one-pot procedure affording ethylmaltol (**1**) at a multigram-scale at ≥0 °C with a markedly improved purity profile. A key advantage of the presented approach to ethylmaltol (**1**) is the use of maltol (**2**) as the starting material, which is readily available from tree barks.

## Supporting Information

File 1 Detailed experimental procedures and characterization data.

## Data Availability

All data that supports the findings of this study is available in the published article and/or the supporting information of this article.
