# Supplementary material for: One-pot synthesis of ethylmaltol from maltol
Source: Beilstein J Org Chem. 2025 Dec 29;21:2755–60. doi: 10.3762/bjoc.21.212 (PMC12766164; doi:10.3762/bjoc.21.212)
Supplement: File 1 — Detailed experimental procedures and characterization data. [file Beilstein_J_Org_Chem-21-2755-s001.pdf]

BEILSTEIN JOURNAL OF ORGANIC CHEMISTRY

Supporting Information

for

One-pot synthesis of ethylmaltol from maltol

Immanuel Plangger, Marcel Jenny, Gregor Plangger and Thomas Magauer

Beilstein J. Org. Chem. **2025,** *21,* 2755–2760. doi:10.3762/bjoc.21.212

Detailed experimental procedures and characterization data

License and Terms: This is a supporting information file under the terms of the Creative Commons Attribution License (https://creativecommons.org/licenses/by/4.0). Please note that the reuse, redistribution and reproduction in particular requires that the author(s) and source are credited and that individual graphics may be subject to special legal provisions.
The license is subject to the *Beilstein Journal of Organic Chemistry* terms and conditions: (https://www.beilstein-journals.org/bjoc/terms)

Table of Contents

1. General experimental details S3
2. Experimental Procedures S5
   - 2.1 Screening of a dianion approach to ethylmaltol (**1**) S5
   - 2.2 Acetyl masking group S7
     - 2.2.1 *O*-(Acetyl)maltol (**8a**) S7
     - 2.2.2 Attempted methylation of *O*-(acetyl)maltol (**8a**) S7
   - 2.3 Carbonate masking group S8
     - 2.3.1 *O*-(Methoxycarbonyl)maltol (**8b**) S8
     - 2.3.2 Attempted methylation of *O*-(methoxycarbonyl)maltol (**8b**) S9
   - 2.4 Methyl masking group S9
     - 2.4.1 *O*-(Methyl)ethylmaltol (**9c**) S9
     - 2.4.2 Ethylmaltol (**1**) from *O*-(methyl)ethylmaltol (**9c**) S10
   - 2.5 Trimethylsilyl masking group S10
     - 2.5.1 *O*-(Trimethylsilyl)maltol (**8d**) S10
     - 2.5.2 Ethylmaltol (**1**) from *O*-(trimethylsilyl)maltol (**8d**) S11
   - 2.6 *tert*-Butyldimethylsilyl masking group S12
     - 2.6.1 *O*-(*tert*-Butyldimethylsilyl)maltol (**8e**) S12
     - 2.6.2 *O*-(*tert*-Butyldimethylsilyl)ethylmaltol (**9e**) S12
     - 2.6.3 Ethylmaltol (**1**) from *O*-(*tert*-butyldimethylsilyl)ethylmaltol (**9e**) S13
     - 2.6.4 Screening of the methylation of *O*-(*tert*-butyldimethylsilyl)maltol (**8e**) S14
     - 2.6.5 One-pot conversion of maltol (**2**) to ethylmaltol (**1**) S15
3. NMR Spectra S22
4. References S26

1. General experimental details

All reactions were performed in oven-dried glassware (110 °C oven temperature) with magnetic stirring under argon atmosphere, unless otherwise noted, using standard Schlenk techniques. If necessary, glassware was further dried under high-vacuum with a heat-gun at 650 °C. Temperature control was performed by external bath thermometers. High temperature reactions were either carried out using a reaction flask connected to a reflux condenser or in sealed pressure tubes while heating with a metal block. Low temperature reactions were either conducted using a distilled water/ice bath (0 °C) or using an acetone bath (Dewar vessel) in combination with an electronically controlled cryostat (−78 °C to 0 °C). Tetrahydrofuran (THF) was dried over molecular sieves (4 Å) prior to use. All other solvents were purchased from Acros Organics (Fisher Scientific) or Sigma Aldrich as 'extra dry' reagents. Solvents for extractions and flash column chromatography (FCC) were purchased in technical grade and purified by distillation prior to use. All reagents were obtained from commercial sources (Sigma Aldrich, Acros Organics (Fisher Scientific), Alfa Aesar, Tokyo Chemical Industry, BLD Pharmatech, Fluorochem, abcr, and ChemPUR) with a purity >95% and used without further purification unless otherwise noted. Maltol (**2**) was purchased from Sigma Aldrich and *O*-methylmaltol (**6a**) was purchased from BLD Pharm and used without further purification. Transfer of air- and moisture-sensitive reagents or solutions was performed under argon atmosphere via syringes through rubber septa. Unless otherwise noted, concentration of reaction mixtures or combined organic layers after extraction was performed on rotary evaporators with a bath temperature of 40 °C. The identity of obtained ethylmaltol (**1**) and maltol (**2**) were confirmed through comparison of the analytical data with those from authentic commercially available samples.

Flash column chromatography (FCC) was carried out using Merck silica gel 60 (0.040–0.063 mm). Analytical thin-layer chromatography (TLC) was carried out using Merck silica gel 60 F254 aluminum foils and visualized under UV light at 254 nm or by staining with either ceric ammonium molybdate (CAM) or an aqueous potassium permanganate ($KMnO_4$) solution and subsequent heating.

High pressure liquid chromatography (HPLC) was conducted on a normal-phase Shimadzu Shim-pack PRC-SIL(H) column (250 × 20 mm, 5 µm particle diameter).

NMR spectra (^{1}H NMR and ^{13}C NMR) were recorded in deuterated chloroform (chloroform-*d*) on a Bruker Avance Neo 400 MHz spectrometer or a 500 MHz spectrometer. For ^{1}H NMR spectra the residual proton peak of the respective solvent (chloroform-*d*: 7.26 ppm) served as internal reference. ^{1}H NMR spectroscopic data is reported as follows: chemical shift δ in ppm (multiplicity, coupling constant *J* in Hz, number of protons). Multiplicities are abbreviated as

follows: s = singlet, d = doublet, t = triplet, q = quartet, p = quintet, h = hextet, br = broad, m = multiplet, or combinations thereof. Combined multiplicities are listed in order of their respective coupling constant *J* starting with the highest one. For ^{13}C NMR the central ^{13}C resonance of the respective solvent (chloroform-*d*: 77.16 ppm) served as internal reference and ^{13}C spectroscopic data is reported as follows: chemical shift δ in ppm (number of carbons in parenthesis if >1). NMR spectra were assigned using information ascertained from COSY, HMBC, HSQC and NOESY experiments. For route exploration and screening, NMR yields were determined by adding 1,1,2,2-tetrachloroethane as an internal standard and recording ^{1}H-NMR spectra using a relaxation delay of 8 s.

High resolution mass spectra (HRMS) were recorded on a Thermo Scientific™ LTQ Orbitrap XL™ Hybrid Ion Trap-Orbitrap Mass Spectrometer at the Institute of Organic Chemistry and Center for Molecular Biosciences, University of Innsbruck.

Infrared spectra (IR) were recorded from 4000 cm^{-1} to 450 cm^{-1} on a Bruker™ ALPHA FT-IR spectrometer. Samples were measured as a neat film by evaporation of a solution in chloroform-*d*. IR data is reported as follows: frequency of absorption in cm^{-1} (absorption intensity), whereby the absorption intensity is abbreviated as follows: w = weak, m = medium, s = strong, br = broad or combinations thereof.

Melting points were measured with a SRS MPA120 EZ-Melt Melting Point Apparatus in open glass capillaries and are uncorrected.

All yields are isolated, unless otherwise specified.

2. Experimental procedures

2.1 Screening of a dianion approach to ethylmaltol (**1**)

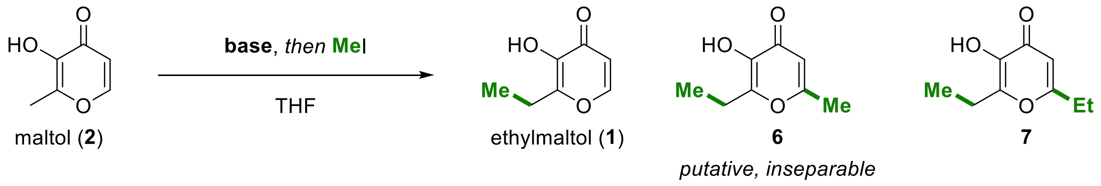

entry	base	temp.	time	yield 1 (6)[1]	yield 7	yield rec. 2
1	NaH, *then n*-BuLi	0 °C → 22 °C	1 h	3%	n.d.	75%
2	MeLi	–20 °C	23 h	11% (2%)	n.d.	2%
3	LDA (2.06 equiv)	0 °C	1.5 h	25%	n.d.	17%
4	LDA (2.20 equiv)	–20 °C	1.5 h	46% (1%)	n.d.	10%
5	LDA (3.00 equiv)	–20 °C	1.5 h	57% (10%)	8%	n.d.
6	LDA (2.15 equiv)	–30 °C	1.5 h	52% (3%)	n.d.	10%

[1]Yield of an inseparable impurity, tentatively identified as **6**, is indicated in brackets.

n.d. = not detected

Procedure for entry 1: To a suspension of sodium hydride (358 mg, 60.0 wt % in mineral oil, 8.96 mmol, 1.13 equiv) in tetrahydrofuran (80.0 mL) was added maltol (**2**) (1.00 g, 7.93 mmol, 1.00 equiv) at 0 °C under an argon atmosphere (**caution: strong gas development**). After the gas evolution ceased (approximately 5 min), the reaction mixture was warmed to 22 °C with a water bath. After stirring for 30 min at 22 °C, the reaction mixture was cooled to 0 °C and a solution of *n*-butyllithium (2.50 M in hexanes, 3.33 mL, 8.33 mmol, 1.05 equiv) was added, whereupon the white suspension turned brownish. After stirring for 10 min at 0 °C, methyl iodide (595 µL, 9.52 mmol, 1.20 equiv) was added. After stirring for additional 10 min at 0 °C, an aqueous saturated solution of ammonium chloride (60 mL) and water (40 mL) were added. The organic layer was separated and the aqueous layer was extracted with ethyl acetate (3 × 80 mL). The combined organic layers were dried over sodium sulfate, the dried solution was filtered, and the filtrate was concentrated under reduced pressure. The residue was purified by flash column chromatography on silica gel (35% to 40% ethyl acetate in cyclohexane) to afford ethylmaltol (**1**) (35.8 mg, 255 µmol, 3%) as a yellowish solid and recovered maltol (**2**) (736 mg, 5.83 mmol, 74%) as a yellowish solid.

Procedure for entry 2: To a solution of maltol (**2**, 1.00 g, 7.93 mmol, 1.00 equiv) in tetrahydrofuran (80.0 mL) was added a solution of methyl lithium (1.60 M in diethyl ether, 5.45 mL, 8.72 mmol, 1.10 equiv) dropwise at −20 °C under an argon atmosphere, during which the colorless solution turned to an orange suspension. After stirring for 5 min at −20 °C, additional methyllithium (1.60 M in diethyl ether, 5.20 mL, 8.33 mmol, 1.05 equiv) was added, whereupon the suspension turned deep purple. After stirring for 22 h at −20 °C, methyl iodide

(1.59 mL, 25.4 mmol, 3.20 equiv) was added, which resulted in the formation of an orange suspension within approximately 2–5 min. After stirring for 25 min at –20 °C, an aqueous saturated solution of ammonium chloride (60 mL) and water (40 mL) were added. The organic layer was separated and the aqueous layer was extracted with ethyl acetate (3 × 80 mL). The combined organic layers were dried over sodium sulfate, the dried solution was filtered, and the filtrate was concentrated under reduced pressure. The residue was purified by flash column chromatography on silica gel (35% to 40% ethyl acetate in cyclohexane) to afford ethylmaltol (**1**) (122 mg, 868 µmol, 11%) contaminated with inseparable, putative 6-(methyl)ethylmaltol (**6**)[1] (21.4 mg, 139 µmol, 2%) as a yellowish solid, recovered maltol (**2**) (23.8 mg, 189 µmol, 2%) as a yellowish solid, and a complex mixture of unidentified side products.

<u>Procedure for entrie 3–6:</u> To a solution of diisopropylamine (equiv as indicated for LDA) in tetrahydrofuran (40.0 mL) was added a solution of *n*-butyllithium (2.50 M in hexanes, equiv as indicated for LDA) at the indicated temperature under an argon atmosphere. After stirring for 15 min, a solution of maltol (**2**) (1.00 g, 7.93 mmol, 1.00 equiv) in tetrahydrofuran (40.0 mL) was added dropwise over 15 min, during which the colorless solution turned first orange and then deep purple. After stirring for 50 min at the indicated temperature, methyl iodide (1.50 equiv for entry 3, 4 and 6, 2.50 equiv for entry 5) was added. After stirring for 30 min at the indicated temperature, an aqueous saturated solution of ammonium chloride (60 mL) and water (40 mL) were added. The organic layer was separated and the aqueous layer was extracted with ethyl acetate (3 × 80 mL). The combined organic layers were dried over sodium sulfate, the dried solution was filtered, and the filtrate was concentrated under reduced pressure. The residue was purified by flash column chromatography on silica gel (30% to 40% ethyl acetate in cyclohexane) to afford 6-(ethyl)ethylmaltol (**7**) as an off-white solid, ethylmaltol (**1**) contaminated with inseparable, putative 6-(methyl)ethylmaltol (**6**) as a yellowish solid and recovered maltol (**2**) as a yellowish solid (yields as indicated in the table).

<u>Analytical data of 6-(ethyl)ethylmaltol (**7**):</u>

TLC (40% ethyl acetate in cyclohexane): R_f = 0.40 (UV, CAM).

mp: 84–85 °C.

^{1}H NMR (400 MHz, $CDCl_3$): δ 6.62 (brs, 1H), 6.20 (t, *J* = 0.7 Hz, 1H), 2.72 (q, *J* = 7.6 Hz, 2H), 2.56 (q, *J* = 7.6 Hz, 2H), 1.26 – 1.20 (m, 6H).

[1] Attempted HPLC purification failed to separate impurity **6** from ethylmaltol (**1**). The identity of **6** was assigned based on NMR studies of the mixture and is supported by isolation and characterization of the side product 6-(ethyl)ethylmaltol (**7**).

^{13}C NMR (101 MHz, $CDCl_3$): δ 174.2, 169.8, 152.0, 140.9, 108.9, 27.2, 21.8, 11.3, 11.1.

IR (ATR, neat): $\tilde{\nu}$ = 3250 (m), 2976 (m), 2940 (w), 2881 (w), 1656 (w), 1616 (s), 1580 (m), 1465 (m), 1421 (w), 1377 (w), 1302 (m), 1250 (w), 1202 (s), 1090 (w), 1066 (w), 1014 (m), 985 (m), 947 (m), 853 (s), 788 (w), 754 (m), 700 (m), 518 (w) cm^{-1}.

HRMS (ESI): calcd for $C_9H_{13}O_3^+$ [M+H]$^+$: 169.0859; found: 169.0854.

2.2 Acetyl masking group

2.2.1 *O*-(Acetyl)maltol (**8a**)

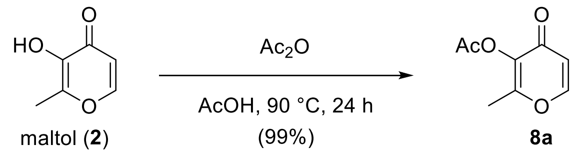

O-(Acetyl)maltol (**8a**) was prepared according to a modified literature procedure [1]: To a vial charged with maltol (**2**) (256 mg, 2.03 mmol, 1.00 equiv) were added in succession acetic anhydride (19.0 mL, 201 mmol, 1.00 equiv) and acetic acid (3.82 mL) at 22 °C under an argon atmosphere. The resulting colorless solution was heated to 90 °C. After stirring for 24 h at 90 °C, the reaction mixture was concentrated under reduced pressure (60 °C, down to 10 mbar) and the remaining yellow oil was diluted with dichloromethane (10 mL) and diethyl ether (10 mL). The organic solution was washed with a saturated aqueous solution of sodium chloride (2 × 20 mL), the washed organic layer was dried over sodium sulfate and the dried solution was filtered. The filtrate was concentrated under reduced pressure to afford *O*-(acetyl)maltol (**8a**) (321 mg, 2.02 mmol, 99%) as a yellow oil.

The obtained analytical data were in accordance with reported literature values [1].

2.2.2 Attempted methylation of *O*-(acetyl)maltol (**8a**)

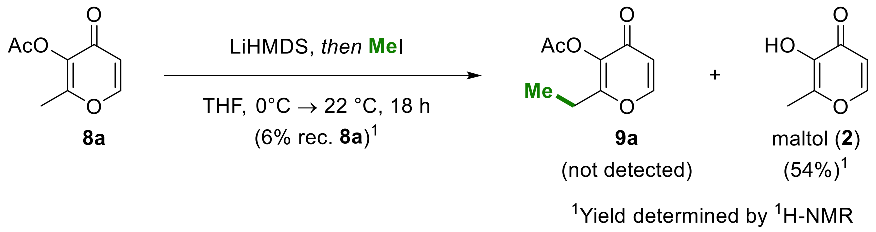

To a solution of *O*-(acetyl)maltol (**8a**) (50.0 mg, 297 µmol, 1.00 equiv) in tetrahydrofuran (1.19 mL) was added a solution of lithium bis(trimethylsilyl)amide (1.00 M in tetrahydrofuran, 357 µL, 357 µmol, 1.20 equiv) at 0 °C under an argon atmosphere, whereupon the colorless

solution turned deep red/purple. After stirring for 30 min at 0 °C, methyl iodide (127 mg, 892 µmol, 3.00 equiv) was added and the reaction mixture was allowed to slowly warm up to 22 °C. After stirring for 18 h, a saturated aqueous solution of ammonium chloride (1 mL) was added to the orange suspension and the biphasic mixture was extracted with ethyl acetate (5 × 1 mL). The combined organic layers were dried over sodium sulfate, the dried solution was filtered, and the filtrate was concentrated under reduced pressure. To the residue was added 1,1,2,2-tetrachloroethane as an NMR standard and the reaction outcome was determined through NMR analysis.

2.3 Carbonate masking group

2.3.1 *O*-(Methoxycarbonyl)maltol (**8b**)

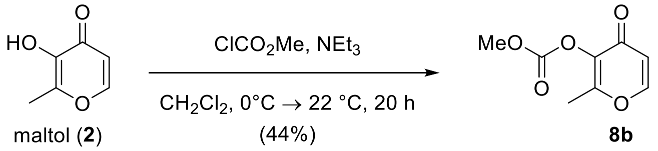

To a suspension of maltol (**2**) (1.00 g, 7.94 mmol, 1.00 equiv) and triethylamine (1.27 mL, 9.13 mmol, 1.15 equiv) in dichloromethane (15.9 mL) was slowly added methyl chloroformate (788 mg, 8.33 mmol, 1.05 equiv) at 0 °C under an argon atmosphere. After stirring for 15 min at 0 °C, the cooling bath was removed and the reaction mixture was allowed to warm up to 22 °C. After stirring for 20 h at 22 °C, the reaction mixture was diluted with ethyl acetate (60 mL) and added to a saturated aqueous solution of ammonium chloride (20 mL) and a saturated aqueous solution of sodium bicarbonate (20 mL). The organic layer was separated and the aqueous layer was extracted with ethyl acetate (20 mL). The combined organic layers were washed in succession with a 1 M aqueous solution of sodium hydroxide (2 × 20 mL), a saturated aqueous solution of sodium bicarbonate (2 × 20 mL), and a saturated aqueous solution of sodium chloride (20 mL). The washed organic layer was dried over sodium sulfate, the dried solution was filtered, and the filtrate was concentrated under reduced pressure to afford *O*-(methoxycarbonyl)maltol (**8b**) (650 mg, 3.53 mmol, 44%) as a white solid.

Analytical data of *O*-(methoxycarbonyl)maltol (**8b**):

TLC (5% methanol in dichloromethane): R_f = 0.52 (UV).

mp: 97–98 °C.

^{1}H NMR (400 MHz, $CDCl_3$): δ 7.67 (d, *J* = 5.7 Hz, 1H), 6.41 (d, *J* = 5.8 Hz, 1H), 3.91 (s, 3H), 2.31 (s, 3H).

^{13}C NMR (101 MHz, $CDCl_3$): δ 171.9, 159.3, 154.4, 152.7, 139.1, 117.1, 56.1, 15.0.

IR (ATR, neat): $\tilde{\nu}$ = 3085 (w), 2962 (w), 1765 (s), 1659 (s), 1637 (s), 1580 (w), 1439 (m), 1424 (m), 1391 (w), 1366 (w), 1269 (s), 1230 (s), 1179 (s), 1084 (w), 1053 (m), 1014 (w), 934 (m), 833 (m), 781 (w), 764 (w), 719 (w), 569 (w), 544 (w), 509 (w), 491 (w) cm^{-1}.

HRMS (ESI): calcd for $C_8H_8NaO_5^+$ $[M+Na]^+$: 207.0264; found: 207.0258.

2.3.2 Attempted methylation of *O*-(methoxycarbonyl)maltol (**8b**)

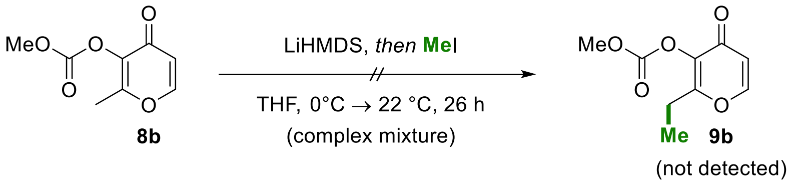

To a solution of *O*-(methoxycarbonyl)maltol (**8b**) (40.4 mg, 219 µmol, 1.00 equiv) in tetrahydrofuran (878 µL) was added a solution of lithium bis(trimethylsilyl)amide (1.00 M in tetrahydrofuran, 263 µL, 263 µmol, 1.20 equiv) at 0 °C under an argon atmosphere, whereupon the colorless solution turned deep purple. After stirring for 30 min at 0 °C, methyl iodide (93.4 mg, 658 µmol, 3.00 equiv) was added and the reaction mixture was allowed to slowly warm up to 22 °C. After stirring for 26 h, a saturated aqueous solution of ammonium chloride (1 mL) was added to the orange suspension and the biphasic mixture was extracted with ethyl acetate (5 × 1 mL). The combined organic layers were dried over sodium sulfate, the dried solution was filtered, and the filtrate was concentrated under reduced pressure. To the residue was added 1,1,2,2-tetrachloroethane as an NMR standard and the reaction outcome was determined through NMR analysis.

2.4 Methyl masking group

2.4.1 *O*-(Methyl)ethylmaltol (**9c**)

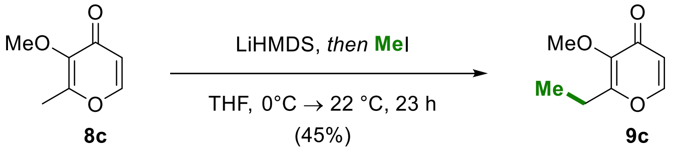

To a solution of *O*-(methyl)maltol (**8c**) (50.6 mg, 95.0 wt %, 343 µmol, 1.00 equiv) in tetrahydrofuran (1.37 mL) was added a solution of lithium bis(trimethylsilyl)amide (1.00 M in tetrahydrofuran, 377 µL, 377 µmol, 1.10 equiv) at 0 °C under an argon atmosphere, whereupon the colorless solution turned deep purple. After stirring for 20 min at 0 °C, methyl

iodide (146 mg, 1.03 mmol, 3.00 equiv) was added and the reaction mixture was allowed to slowly warm up to 22 °C. After stirring for 22.5 h, a saturated aqueous solution of ammonium chloride (1 mL) was added to the orange suspension and the biphasic mixture was extracted with ethyl acetate (5 × 1 mL). The combined organic layers were dried over sodium sulfate, the dried solution was filtered, and the filtrate was concentrated under reduced pressure. The residue was purified by flash column chromatography on silica gel (50% to 60% ethyl acetate in cyclohexane) to afford *O*-(methyl)ethylmaltol (**9c**) (23.8 mg, 154 µmol, 45%) as a yellowish oil.

The obtained analytical data were in accordance with reported literature values [2].

2.4.2 Ethylmaltol (**1**) from *O*-(methyl)ethylmaltol (**9c**)

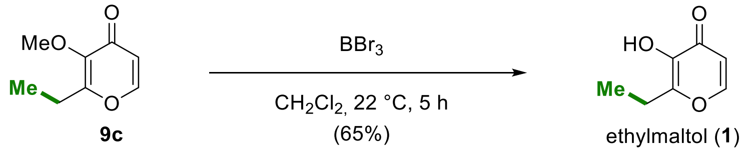

To a solution of *O*-(methyl)ethylmaltol (**9c**) (10.0 mg, 64.9 µmol, 1.00 equiv) in dichloromethane (500 µL) was added a solution of boron tribromide (1.00 M in dichloromethane, 324 µL, 324 µmol, 5.00 equiv) at 22 °C under an argon atmosphere, whereupon the yellowish solution turned red. After stirring for 5 h at 22 °C, a saturated aqueous solution of sodium bicarbonate (1 mL) was added to the orange suspension and the biphasic mixture was extracted with ethyl acetate (6 × 1 mL). The combined organic layers were dried over sodium sulfate, the dried solution was filtered, and the filtrate was concentrated under reduced pressure. The residue was purified by flash column chromatography on silica gel (35% to 40% ethyl acetate in cyclohexane) to afford ethylmaltol (**1**) (5.9 mg, 42 µmol, 65%) as an off-white solid.

2.5 Trimethylsilyl masking group

2.5.1 *O*-(Trimethylsilyl)maltol (**8d**)

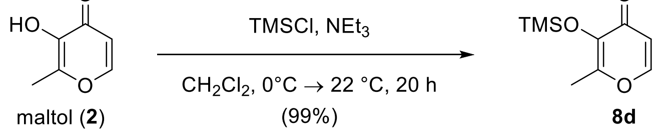

To a suspension of maltol (**2**) (1.00 g, 7.93 mmol, 1.00 equiv) and triethylamine (1.66 mL, 11.9 mmol, 1.50 equiv) in dichloromethane (15.0 mL) was slowly added chlorotrimethylsilane

(788 mg, 8.33 mmol, 1.05 equiv) at 0 °C under an argon atmosphere. After complete addition, the cooling bath was removed and the reaction mixture was allowed to warm up to 22 °C. After stirring for 20 h at 22 °C, the reaction mixture was diluted with ethyl acetate (50 mL) and washed with a saturated aqueous solution of sodium chloride (2 × 20 mL). The washed organic layer was dried over sodium sulfate, the dried solution was filtered, and the filtrate was concentrated under reduced pressure to afford *O*-(trimethylsilyl)maltol (**8d**) (1.55 g, 7.82 mmol, 99%) as an orange oil.

Analytical data of *O*-(trimethylsilyl)maltol (**8d**):

TLC (5% methanol in dichloromethane): R_f = 0.45 (UV).

^{1}H NMR (400 MHz, $CDCl_3$): δ 7.57 (d, *J* = 5.6 Hz, 1H), 6.29 (d, *J* = 5.5 Hz, 1H), 2.26 (s, 3H), 0.25 (s, 9H).

^{13}C NMR (101 MHz, $CDCl_3$): δ 174.4, 155.1, 153.0, 142.7, 115.8, 14.7, 1.3 (3C).

IR (ATR, neat): $\tilde{\nu}$ = 3248 (m), 3062 (m), 2923 (w), 1654 (m), 1615 (s), 1559 (m), 1457 (m), 1397 (m), 1370 (m), 1287 (s), 1254 (s), 1222 (s), 1197 (m), 1074 (m), 1022 (m), 953 (w), 916 (m), 839 (s), 760 (w), 688 (s), 562 (m), 506 (s) cm^{-1}.

HRMS (ESI): calcd for $C_9H_{14}NaO_3Si^+$ $[M+Na]^+$: 221.0604; found: 221.0604.

2.5.2 Ethylmaltol (**1**) from *O*-(trimethylsilyl)maltol (**8d**)

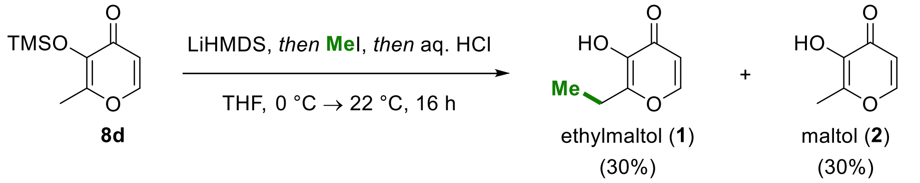

To a solution of *O*-(trimethylsilyl)maltol (**8d**) (100 mg, 504 µmol, 1.00 equiv) in tetrahydrofuran (2.02 mL) was added a solution of lithium bis(trimethylsilyl)amide (1.00 M in tetrahydrofuran, 555 µL, 555 µmol, 1.10 equiv) at 0 °C under an argon atmosphere, whereupon the colorless solution turned deep blue. After stirring for 30 min at 0 °C, methyl iodide (94.6 µL, 1.51 mmol, 3.00 equiv) was added and the reaction mixture was allowed to slowly warm up to 22 °C. After stirring for 14.5 h, an aqueous solution of hydrochloric acid (1.00 M, 3.03 mL, 3.03 mmol, 6.00 equiv) was added. After stirring for 1 h at 22 °C, the reaction mixture was extracted with ethyl acetate (4 × 2 mL), the combined organic layers were dried over sodium sulfate, the dried solution was filtered, and the filtrate was concentrated under reduced pressure. The residue was purified by flash column chromatography on silica gel (35% to 40% ethyl acetate

in cyclohexane) to afford ethylmaltol (**1**) (21.3 mg, 152 µmol, 30%) as an off-white solid and maltol (**2**) (19.0 mg, 151 µmol, 30%) as an off-white solid.

2.6 *tert*-Butyldimethylsilyl masking group

2.6.1 *O*-(*tert*-Butyldimethylsilyl)maltol (**8e**)

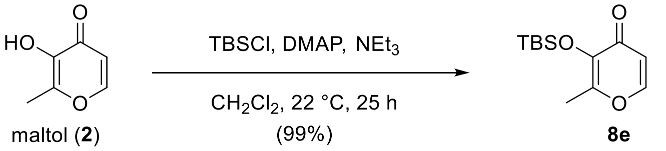

O-(*tert*-Butyldimethylsilyl)maltol (**8e**) was prepared according to a modified literature procedure [3,4]: To a suspension of maltol (**2**, 2.00 g, 15.9 mmol, 1.00 equiv), triethylamine (2.43 mL, 17.6 mmol, 1.10 equiv), and 4-dimethylaminopyridine (DMAP, 58.2 mg, 476 µmol, 3.00 mol %) in dichloromethane (30.0 mL) was slowly added *tert*-butylchlorodimethylsilane (2.44 g, 16.2 mmol, 1.02 equiv) at 22 °C under an argon atmosphere. After stirring for 25 h at 22 °C, the reaction mixture was poured into a mixture of water (25 mL) and a saturated aqueous solution of ammonium chloride (25 mL). The organic layer was separated and the aqueous layer was extracted with dichloromethane (2 × 10 mL). The combined organic layers were dried over sodium sulfate, the dried solution was filtered, and the filtrate was concentrated under reduced pressure to afford *O*-(*tert*-butyldimethylsilyl)maltol (**8e**, 3.76 g, 15.7 mmol, 99%) as a white solid.

The obtained analytical data were in accordance with reported literature values [3,4].

2.6.2 *O*-(*tert*-Butyldimethylsilyl)ethylmaltol (**9e**)

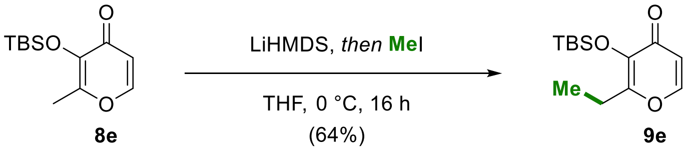

To a solution of *O*-(*tert*-butyldimethylsilyl)maltol (**8e**) (49.1 mg, 204 µmol, 1.00 equiv) in tetrahydrofuran (817 µL) was added a solution of lithium bis(trimethylsilyl)amide (1.00 M in tetrahydrofuran, 225 µL, 225 µmol, 1.10 equiv) at 0 °C under an argon atmosphere, whereupon the colorless solution turned deep violet. After stirring for 30 min at 0 °C, methyl iodide (87.0 mg, 613 µmol, 3.00 equiv) was added. After stirring for 16 h at 0 °C, a saturated aqueous solution of ammonium chloride (1 mL) was added and the resulting biphasic mixture

was extracted with ethyl acetate (5 × 1 mL). The combined organic layers were dried over sodium sulfate, the dried solution was filtered, and the filtrate was concentrated under reduced pressure. The residue was purified by flash column chromatography on silica gel (10% to 20% ethyl acetate in cyclohexane) to afford *O*-(*tert*-butyldimethylsilyl)ethylmaltol (**9e**) (33.3 mg, 131 µmol, 64%) as a colorless oil.

Analytical data of *O*-(*tert*-butyldimethylsilyl)ethylmaltol (**9e**):

TLC (20% ethyl acetate in cyclohexane): R_f = 0.45 (UV, CAM).

^{1}H NMR (500 MHz, $CDCl_3$): δ 7.61 (d, *J* = 5.6 Hz, 1H), 6.29 (d, *J* = 5.5 Hz, 1H), 2.71 (q, *J* = 7.6 Hz, 2H), 1.21 (t, *J* = 7.5 Hz, 3H), 0.96 (s, 9H), 0.27 (s, 6H).

^{13}C NMR (126 MHz, $CDCl_3$): δ 174.5, 159.0, 153.0, 142.2, 115.6, 26.1 (3C), 22.0, 18.9, 11.2, –3.6 (2C).

IR (ATR, neat): $\tilde{\nu}$ = 2954 (w), 2930 (w), 2885 (w), 2857 (w), 1643 (s), 1575 (w), 1464 (w), 1427 (w), 1387 (w), 1324 (w), 1257 (s), 1205 (m), 1100 (w), 1066 (w), 988 (w), 938 (w), 885 (m), 825 (s), 785 (m), 678 (w), 576 (w), 535 (w), 513 (w), 455 (w) cm^{-1}.

HRMS (ESI): calcd for $C_{13}H_{22}NaO_3Si^+$ $[M+Na]^+$: 277.1230; found: 277.1220.

2.6.3 Ethylmaltol (**1**) from *O*-(*tert*-butyldimethylsilyl)ethylmaltol (**9e**)

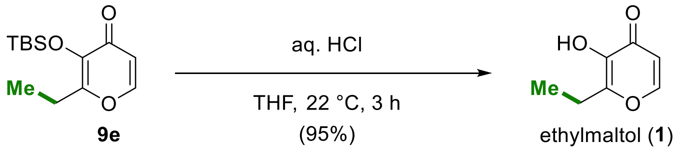

To a solution of *O*-(*tert*-butyldimethylsilyl)ethylmaltol (**9e**, 30.5 mg, 120 µmol, 1.00 equiv) in tetrahydrofuran (1.00 mL) was added an aqueous solution of hydrochloric acid (2.00 M, 1.20 mL, 2.40 mmol, 20.0 equiv) at 22 °C, whereupon the colorless solution turned yellow. After stirring for 3 h at 22 °C, water (2 mL) was added and the biphasic mixture was extracted with ethyl acetate (5 × 2 mL). The combined organic layers were dried over sodium sulfate, the dried solution was filtered, and the filtrate was concentrated under reduced pressure. The residue was purified by flash column chromatography on silica gel (35% to 40% ethyl acetate in cyclohexane) to afford ethylmaltol (**1**, 16.0 mg, 114 µmol, 95%) as an off-white solid.

2.6.4 Screening of methylation conditions of *O*-(*tert*-butyldimethylsilyl)maltol (**8e**)

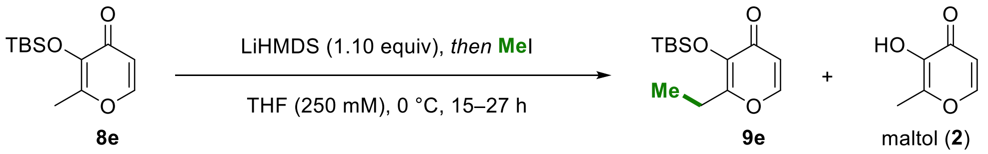

entry	deviation	NMR yield 9e	8e	2
1	none	64%[1]	n.d.	n.d.
2	0 °C → 22 °C (slow warm-up)	59%	n.d.	n.d.
3	22 °C	46%	n.d.	n.d.
4	2-Me-THF	37%	n.d.	n.d.
5	NaHMDS (1.10 equiv), 0 °C → 22 °C	51%	n.d.	n.d.
6	NaH (1.10 equiv), HMDS (12.0 mol%), 22 °C	6%	71%	n.d.
7	LiO*t*-Bu (1.20 equiv), 0 °C → 22 °C	5%	76%	n.d.
8	Mg(O*t*-Bu)$_2$ (2.30 equiv), 0 °C → 22 °C	n.d.	57%	37%
9	NaO*t*-Bu (1.20 equiv), 0 °C → 22 °C	n.d.	11%	75%
10	KO*t*-Bu (2.00 equiv), 0 °C → 22 °C	n.d.	n.d.	24%
11	DBU (2.00 equiv), 0 °C → 22 °C	n.d.	95%	n.d.

[1]Isolated yield

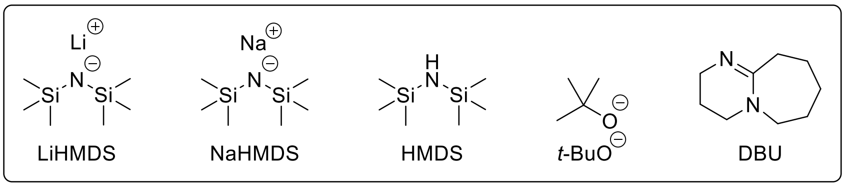

General procedure: To a solution of *O*-(*tert*-butyldimethylsilyl)maltol (**8e**, 204 µmol, 1.00 equiv) in tetrahydrofuran (250 mM) were added the indicated reagents in the indicated stoichiometry at 0 °C (for entries 3 and 6: 22 °C). For lithium bis(trimethylsilyl)amide (LiHMDS) and sodium bis(trimethylsilyl)amide (NaHMDS), a 1.00 M solution in tetrahydrofuran was used. After stirring for 30 min at 0 °C (entries 3 and 6: 22 °C), methyl iodide (613 µmol, 3.00 equiv) was added and stirring was either continued at 0 °C (entries 1 and 4), at 22 °C (entries 3 and 6), or the reaction mixture was allowed to slowly warm up to 22 °C. After stirring for 15–27 h at the indicated temperature, a saturated aqueous solution of ammonium chloride (1 mL) was added and the resulting biphasic mixture was extracted with ethyl acetate (5 × 1 mL). The combined organic layers were dried over sodium sulfate, the dried solution was filtered, and the filtrate was concentrated under reduced pressure. To the residue was added 1,1,2,2-tetrachloroethane as an NMR standard and the yield was determined through NMR analysis. For entry 1, purification via flash column chromatography is described in section 2.6.2.

2.6.5 One-pot conversion of maltol (**2**) to ethylmaltol (**1**)

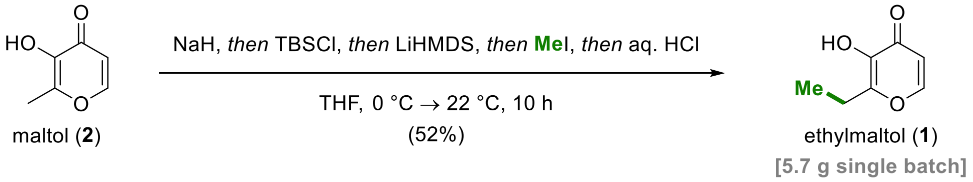

To a suspension of sodium hydride (3.58 g, 60.0 wt% in mineral oil, 89.6 mmol, 1.13 equiv) in tetrahydrofuran (317 mL) was added maltol (**2**) (10.0 g, 79.3 mmol, 1.00 equiv) at 0 °C under an argon atmosphere (**caution: strong gas development**). After the gas evolution ceased (approximately 10 min), the reaction mixture was warmed to 22 °C with a water bath. After stirring for 70 min at 22 °C, *tert*-butylchlorodimethylsilane (13.5 g, 89.6 mmol, 1.13 equiv) was added and stirring was continued for 7 h at 22 °C. The reaction mixture was cooled to 0 °C and a solution of lithium bis(trimethylsilyl)amide (1.00 M in tetrahydrofuran, 95.3 mL, 95.2 mmol, 1.20 equiv) was added over 5 min, whereupon the off-white suspension turned deep purple.[2] After stirring for 8 min at 0 °C, methyl iodide (14.9 mL, 238 mmol, 3.00 equiv) was rapidly added. After stirring for a further 1 h at 0 °C, an aqueous solution of hydrochloric acid (2.00 M, 79.0 mL, 158 mmol, 1.99 equiv)[3] was added portionwise to adjust the pH to ≈1–2. Upon complete silyl deprotection as indicated by thin layer chromatography (TLC) (approx. 15–30 min), a saturated aqueous solution of sodium bicarbonate (20 mL) was added to adjust the pH to ≈6–7. The organic layer was separated and the aqueous layer was extracted with ethyl acetate (3 × 100 mL). The combined organic layers were dried over sodium sulfate, the dried solution was filtered, and the filtrate was concentrated under reduced pressure. The residue was purified by flash column chromatography on silica gel (35% to 40% ethyl acetate in cyclohexane) to afford ethylmaltol (**1**, 5.74 g, 41.0 mmol, 52%) as an off-white/yellowish solid.[4]

<u>Decolorization of ethylmaltol (**1**) by vacuum distillation:</u>

A 100 mL round-bottom flask charged with off-white/yellowish ethylmaltol (**1**) (3.13 g, 22.3 mmol) was attached to a cold finger with a dry ice trap. During vacuum distillation (0.5–0.6 mbar) with heating in an oil bath at 130–150 °C, ethylmaltol (**1**) first melted and then pure

---

[2] An alternative order of addition, i.e., transferring the generated *O*-(*tert*-butyldimethylsilyl) maltol (**8e**) solution via cannulation to the solution of lithium bis(trimethylsilyl)amide in tetrahydrofuran, afforded comparable results.

[3] A range of 1.77–1.99 equiv of an aqueous solution of hydrochloric acid provided comparable results. Addition of 5.00 equiv of an aqueous solution of hydrochloric acid led to decomposition.

[4] While the obtained ethylmaltol (**1**) was pure based on standard analytic techniques (NMR, melting point), the off-white/yellowish appearance differed when compared to commercial samples. Recrystallization from ethanol and chloroform failed to provide pure white ethylmaltol (**1**).

white ethylmaltol (**1**) (2.75 g, 19.6 mmol, 88% recovery) was deposited on the cold finger (see also Figure S1 and S2).

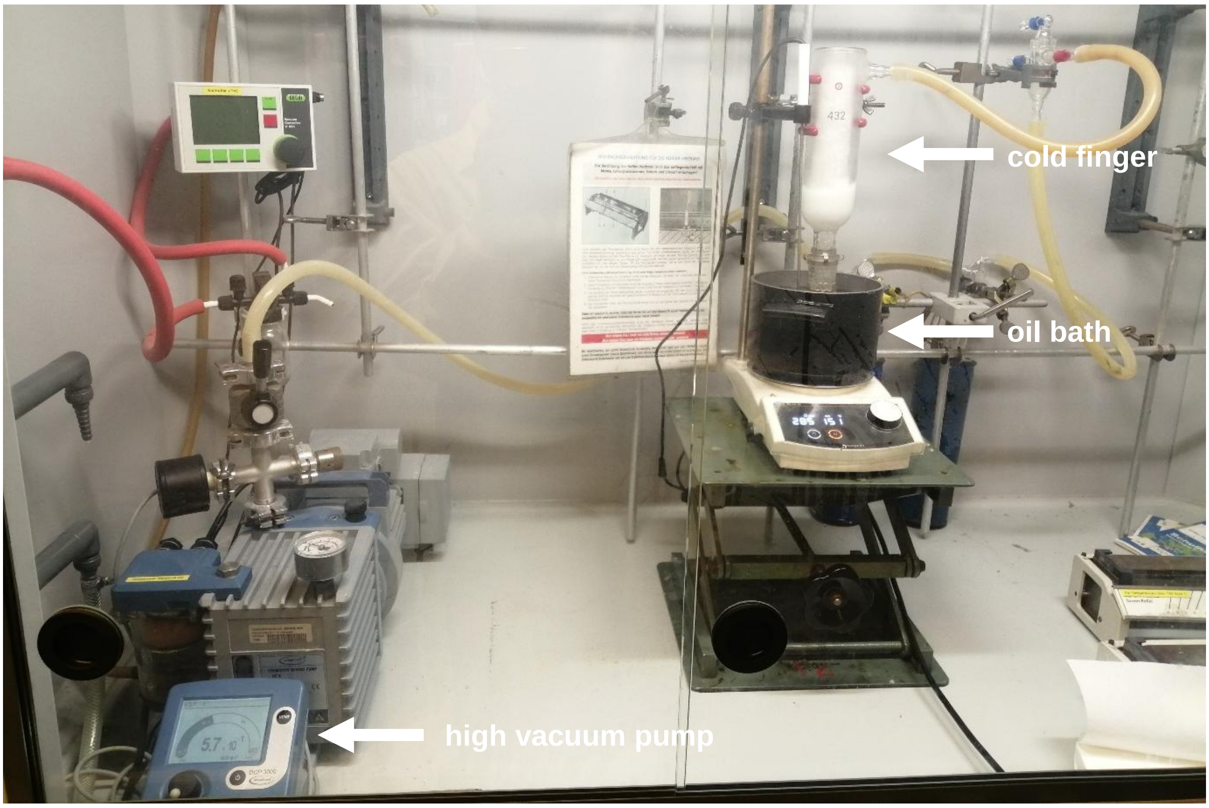

Figure S1. Vacuum distillation set-up to obtain decolorized, white ethylmaltol (**1**).

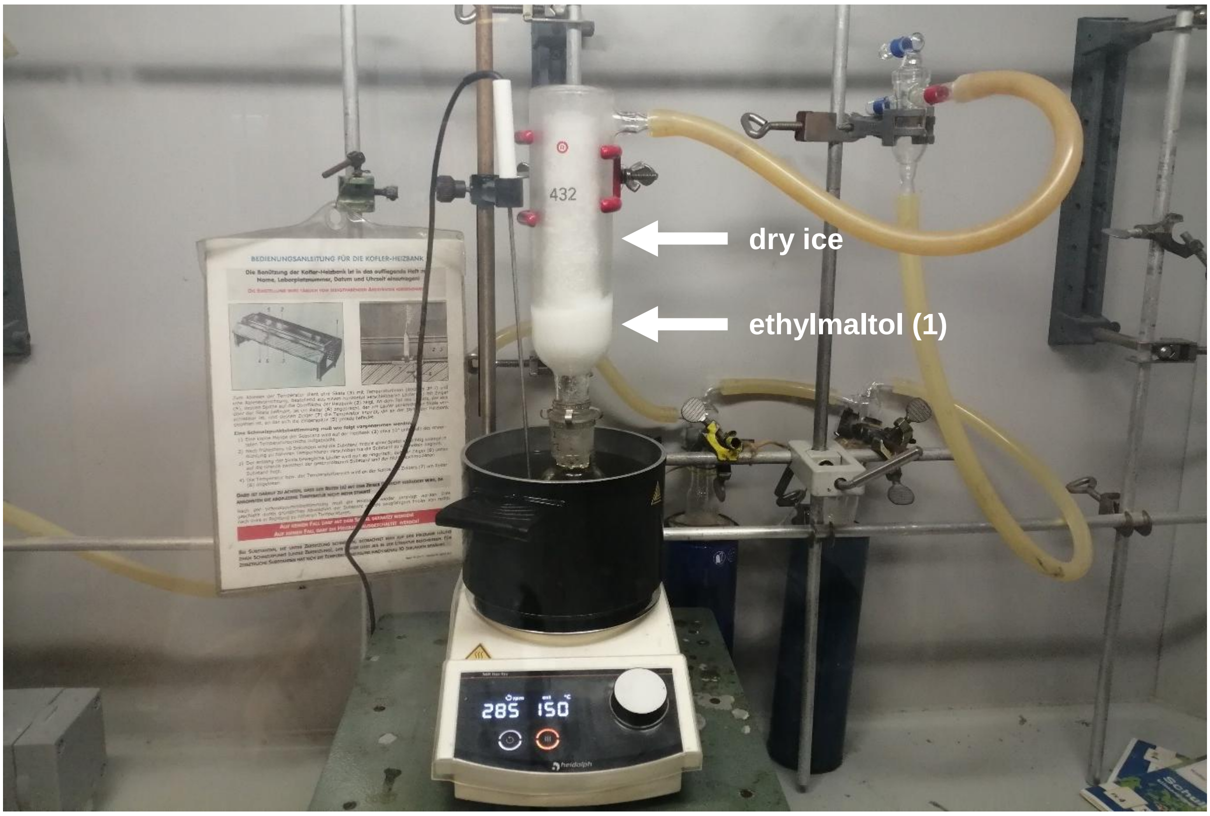

Figure S2. White ethylmaltol (**1**) is deposited on the cold finger.

^{1}H NMR spectrum of ethylmaltol (**1**) and physical appearance:

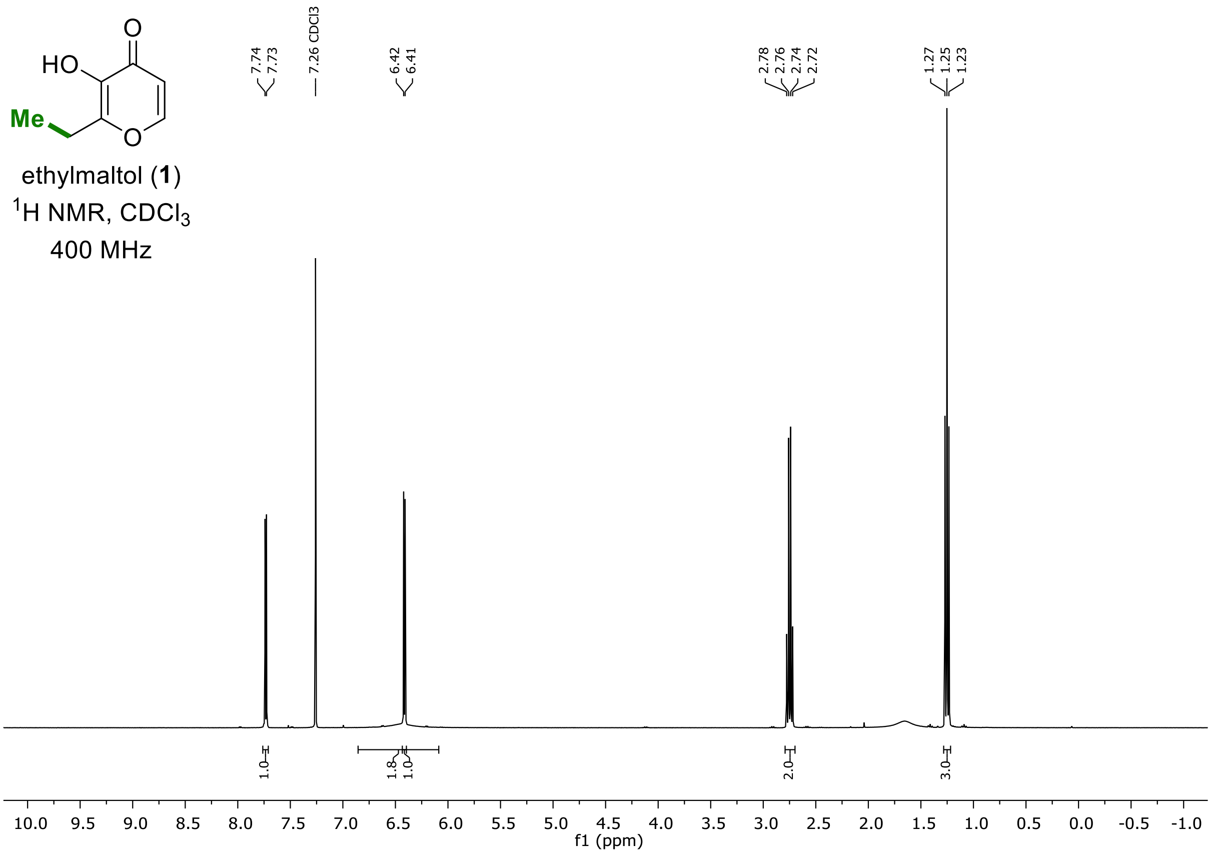

^{1}H NMR (400 MHz, CDCl$_3$): δ 7.73 (d, *J* = 5.5 Hz, 1H), 6.86 – 6.09 (brs, 1H), 6.41 (d, *J* = 5.5 Hz, 1H), 2.75 (q, *J* = 7.6 Hz, 2H), 1.25 (t, *J* = 7.6 Hz, 3H).

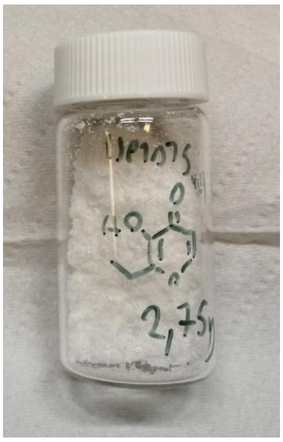

Figure S3. White ethylmaltol (**1**) obtained from vacuum distillation.

Analytical characterization of ethylmaltol (**1**):

The synthesized ethylmaltol was subjected to comprehensive analytical characterization. The evaluation was conducted in accordance with the specifications and permissible limits outlined in the Food Chemicals Codex (FCC), effective as of March 22, 2022, and the United States Pharmacopeia–National Formulary (USP–NF), effective as of July 10, 2024. In addition to standard quality parameters, supplementary analyses were performed to quantify chloride levels and to screen for cationic impurities other than heavy metals, with the aim of identifying potential contaminants originating from the synthesis process. The detected concentrations of these impurities were found to be within acceptable limits and do not pose a concern from a food safety perspective.

Appearance The physical characteristics of the ethylmaltol powder were assessed through visual inspection and basic handling. The sample was examined for color, texture, and homogeneity. It appeared as a fine, white to slightly off-white crystalline powder, consistent with the description provided in the FCC. No visible impurities or discoloration were observed.

Solubility 200 milligrams of the sample were dissolved in 11 mL of water. The resulting solution was visually inspected and found to be clear, indicating compliance with FCC requirements for solubility and clarity.

Ash 500 milligrams of the sample were incinerated in a suitable crucible at 600 °C until complete combustion of organic matter was achieved. The remaining inorganic residue was weighed and expressed as a percentage of the original sample mass.

Water content The water content of ethylmaltol was measured using Karl Fischer titration, in accordance with standard procedures for moisture analysis. 200 milligrams of sample were introduced into the titration vessel, and the water content was quantified by volumetric Karl Fischer reagent consumption. The result was expressed as a percentage of water by weight

Melting point The melting point of ethylmaltol was determined using a capillary melting point apparatus (Mettler Toledo MP70 Melting Point System). A small amount of the sample was packed into a capillary tube and heated under controlled conditions. The temperature range over which the sample transitioned from solid to liquid was recorded. The observed melting point was compared to the specification listed in the Food Chemicals Codex (FCC) to confirm identity and purity.

Cations The presence of heavy metals and other cations was analyzed using Inductively Coupled Plasma Optical Emission Spectrometry (Agilent Technologies 5100 ICP-OES). 200 milligrams of the sample were digested using 69% nitric acid at 180 °C for 30 minutes, to ensure complete dissolution of inorganic constituents. The resulting solution was introduced

into the ICP-OES instrument, where elemental concentrations were quantified based on their characteristic emission spectra. The analysis included both heavy metals and additional cations potentially introduced during synthesis. Results were evaluated against a multi-element standard (ROTI®Star multi element ICP standard solution).

Chloride Chloride content was quantified using ion chromatography (Metrohm 940 Professional IC Vario). 100 milligrams of the sample were dissolved in 50 mL deionized water and filtered to remove particles. The solution was injected into the ion chromatograph equipped with a conductivity detector (Metrohm IC conductivity detector) and an anion exchange column (Metrosep A Supp 5 µm 150 × 4.0 mm). Chloride ions were separated and detected based on their retention time and conductivity response. Quantification was performed using calibration standards (*Trace***CERT**®, 1000 mg/L chloride in water).

Identification Ethylmaltol was identified using Fourier transform near-infrared (FT-NIR) spectroscopy (MPA, Bruker). A representative sample was scanned in the near-infrared region, and the resulting spectrum was compared to a reference standard. Characteristic absorption bands corresponding to functional groups present in ethylmaltol were evaluated to confirm identity. The spectral match confirmed the presence and purity of ethylmaltol in accordance with established criteria.

Assay High-performance liquid chromatography (HPLC) analyses of ethylmaltol was performed, using a Shimadzu LC-20AD HPLC with a photodiode-array detector (SPD-M20A), autosampler, and a reversed phase Kinetex C-18 column (5 µm , 100 Å, 150 × 4,6 mm) in an isocratic elution mode with the mobile phase water/formic acid (A) (0.1% formic acid in water)/acetonitrile (B) (90:10, v/v). The mobile phase was degassed using an ultrasonic bath and the run time was 12 minutes, with a flow rate of 1 mL/min. The column temperature was set at 40 °C, and the analytes were detected at 274 nm. Ethylmaltol was quantified using a calibration curve established with the corresponding standard (Sigma-Aldrich, ethylmaltol <99%). 10 milligrams of the sample were dissolved in 250 mL deionized water and filtered to remove particles; each analysis was performed in duplicate.

Parameters	Lower Limit	Upper Limit	Result	Specification
Appearance			white, crystalline powder	FCC
Solubility			soluble in water	FCC
Ash [%]		0.2	0.02 ± 0.0	USP-NF
Water content [%]		0.5	0.01± 0.0	USP-NF
Melting point [°C]	88	92	91.5 ± 0.2	FCC*
Identification			ok	FCC
Assay [%]	97.5	102.5	98.5 ± 0.7	USP-NF
Arsenic [mg/kg]		1.00	< 0.05	Internal req.
Lead [mg/kg]		1.00	0.46 ± 0.07	Internal req.
Mercury [mg/kg]		1.00	< 0.05	Internal req.
Cadmium [mg/kg]		1.00	< 0.05	Internal req.
Aluminum [mg/kg]			0.69 ± 0.14	
Barium [mg/kg]			0.19 ± 0.23	
Bismuth [mg/kg]			2.13 ± 1.15	
Calcium [mg/kg]			40.29 ± 0.20	
Iron [mg/kg]			0.99 ± 0.42	
Potassium [mg/kg]			1.00 ± 0.18	
Lithium [mg/kg]			0.20 ± 0.01	
Magnesium [mg/kg]			1.51 ± 0.13	
Sodium [mg/kg]			3.41 ± 0.18	
Nickel [mg/kg]			0.15 ± 0.01	
Phosphorous [mg/kg]			0.50 ± 0.11	
Sulphur [mg/kg]			12.49 ± 0.18	
Antimony [mg/kg]			0.75 ± 0.08	
Selenium [mg/kg]			1.13 ± 0.07	
Tin [mg/kg]			0.11 ± 0.02	
Zinc [mg/kg]			1.20 ± 0.18	
Chloride [mg/kg]			< 5.0	

Table S1: Analytical characterization of ethylmaltol. The results show the mean ± standard deviation from two independent measurements of the sample. Cation screening performed via ICP-OES indicated that boron, beryllium, manganese, molybdenum, strontium, thallium, and vanadium were below the limit of quantification (LOQ = 0.05 mg/kg). ***** internal specification based on FCC (about 90 °C).

3. NMR spectra

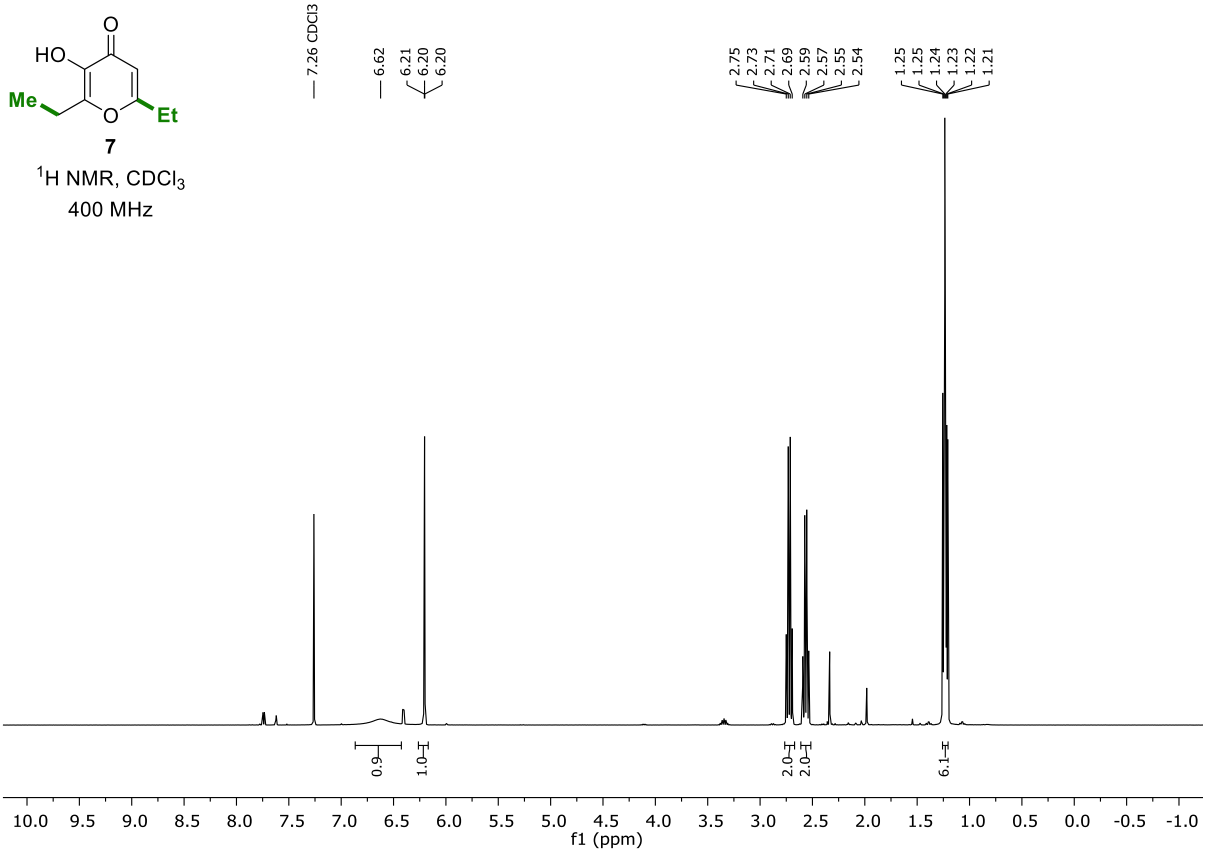

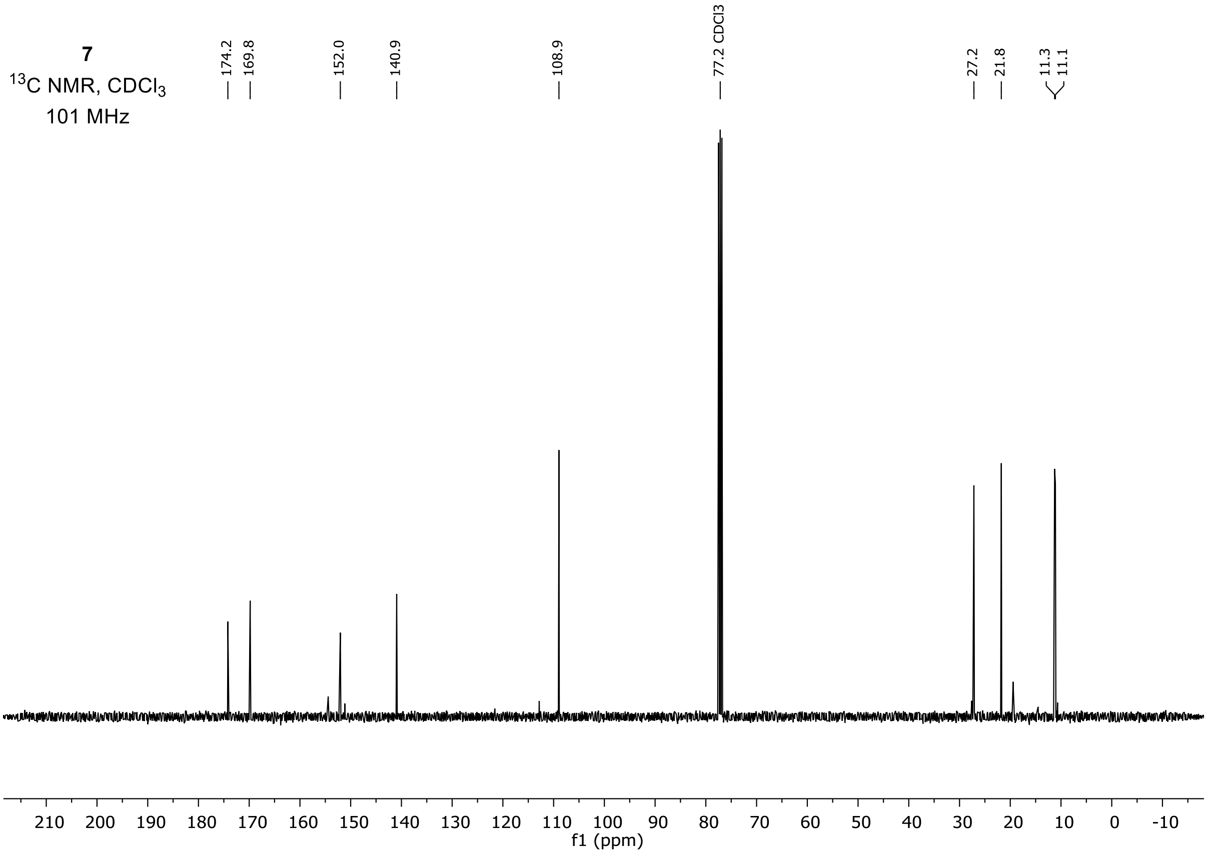

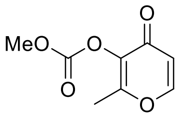

8b

^1H NMR, $CDCl_3$

400 MHz

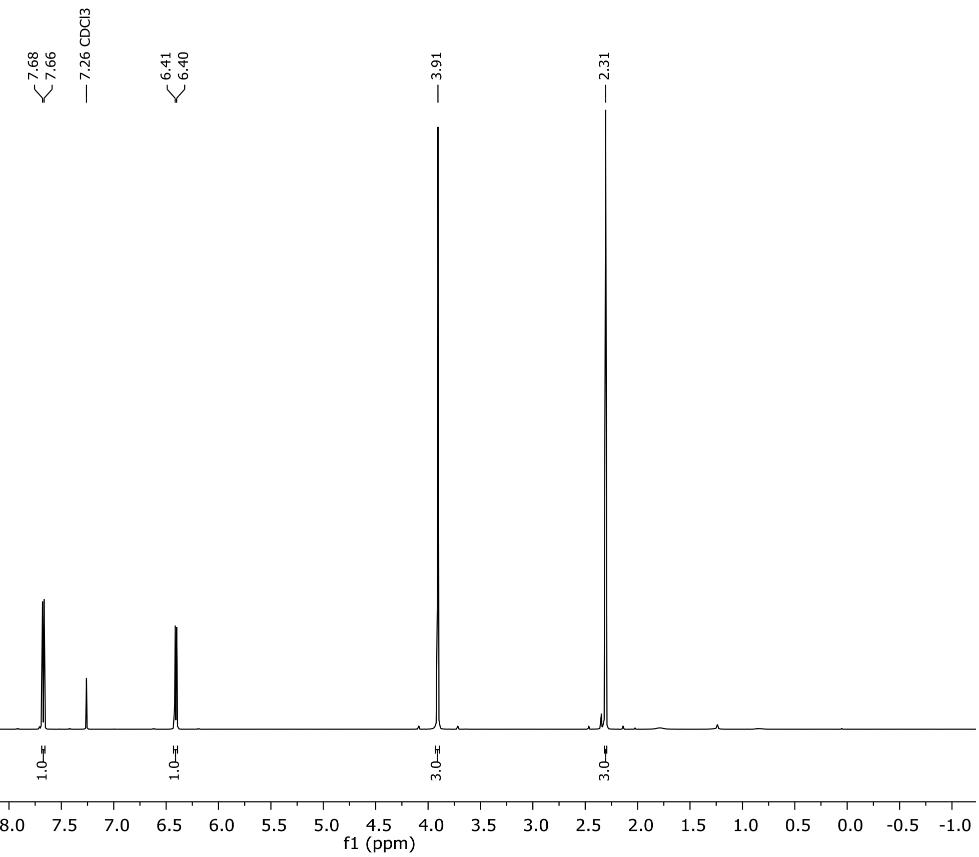

8b

^{13}C NMR, $CDCl_3$

101 MHz

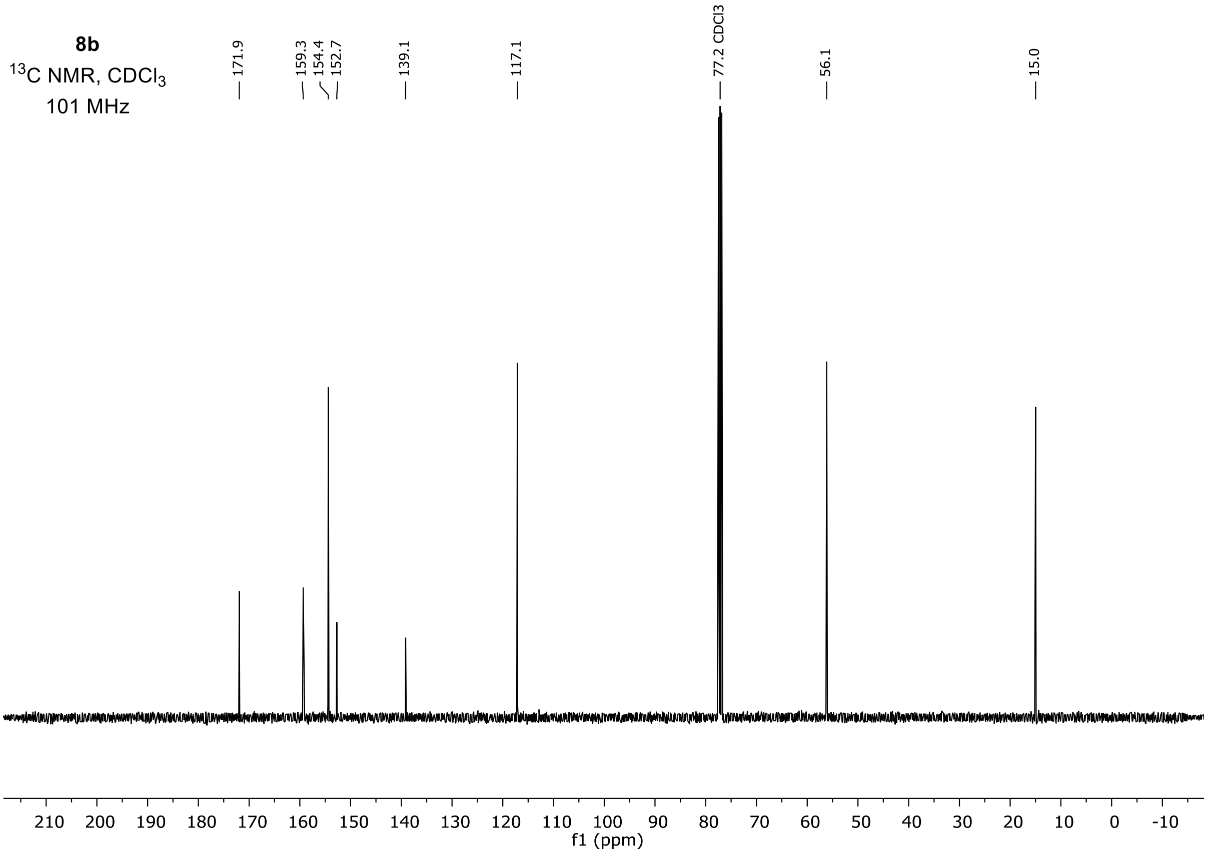

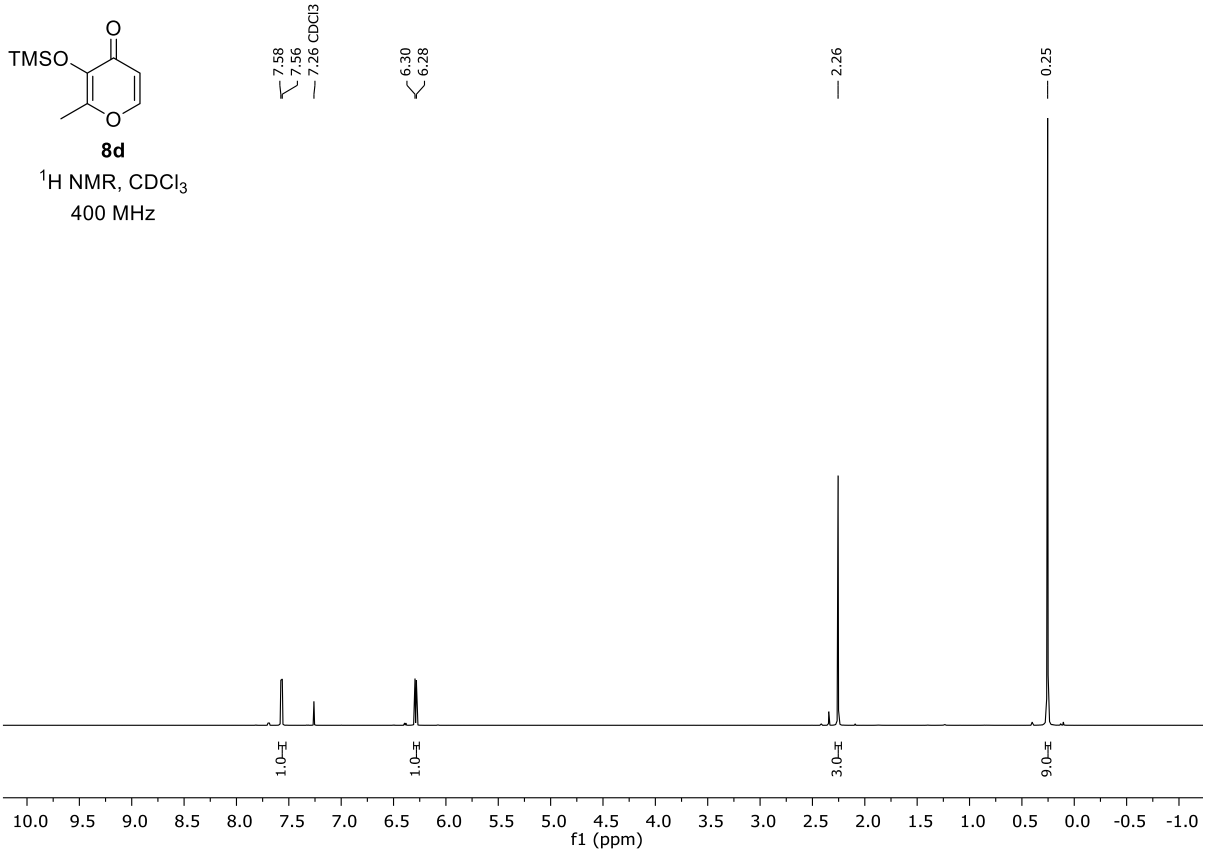
TMSO
O
O
8d
¹H NMR, CDCl₃
400 MHz
7.58
7.56
7.26 CDCl3
6.30
6.28
2.26
0.25
1.0
1.0
3.0
9.0
10.0 9.5 9.0 8.5 8.0 7.5 7.0 6.5 6.0 5.5 5.0 4.5 4.0 3.5 3.0 2.5 2.0 1.5 1.0 0.5 0.0 -0.5 -1.0
f1 (ppm)

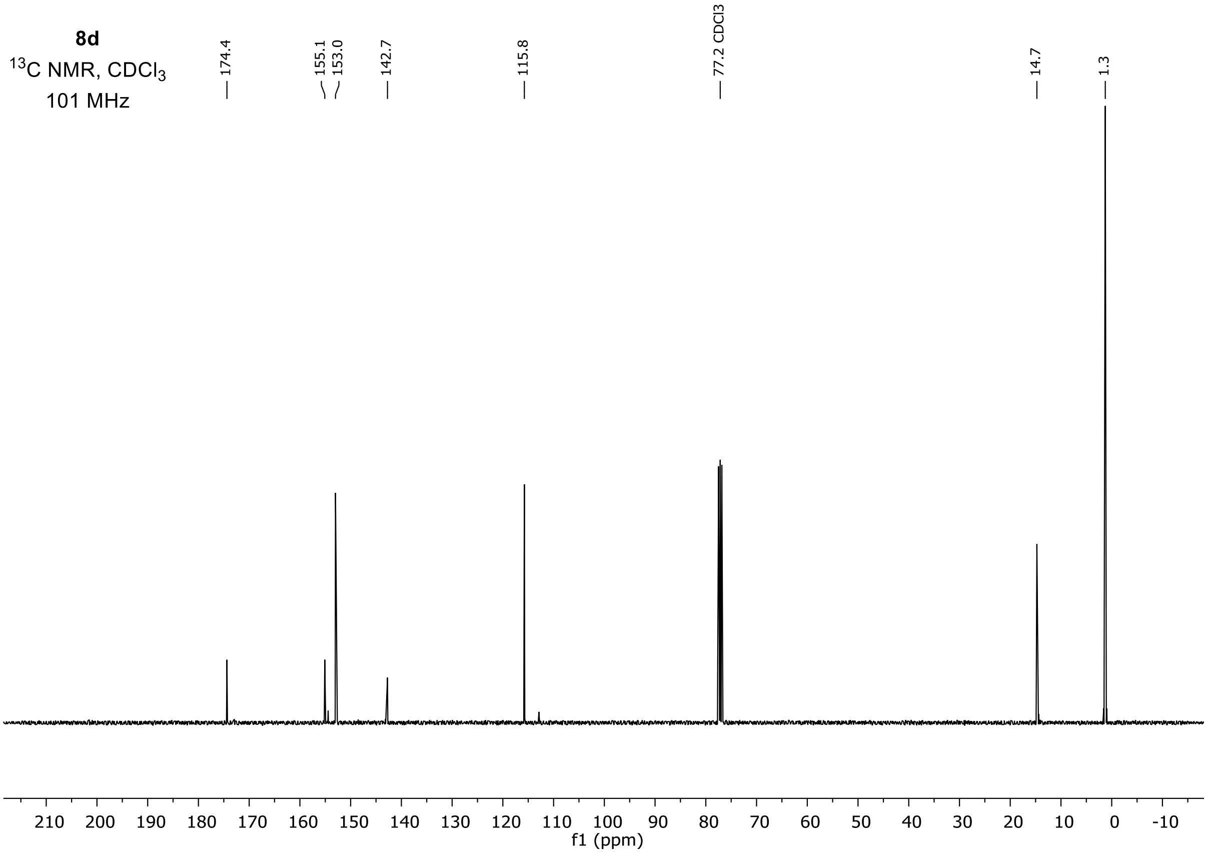
8d
¹³C NMR, CDCl₃
101 MHz
174.4
155.1
153.0
142.7
115.8
77.2 CDCl3
14.7
1.3
210 200 190 180 170 160 150 140 130 120 110 100 90 80 70 60 50 40 30 20 10 0 -10
f1 (ppm)

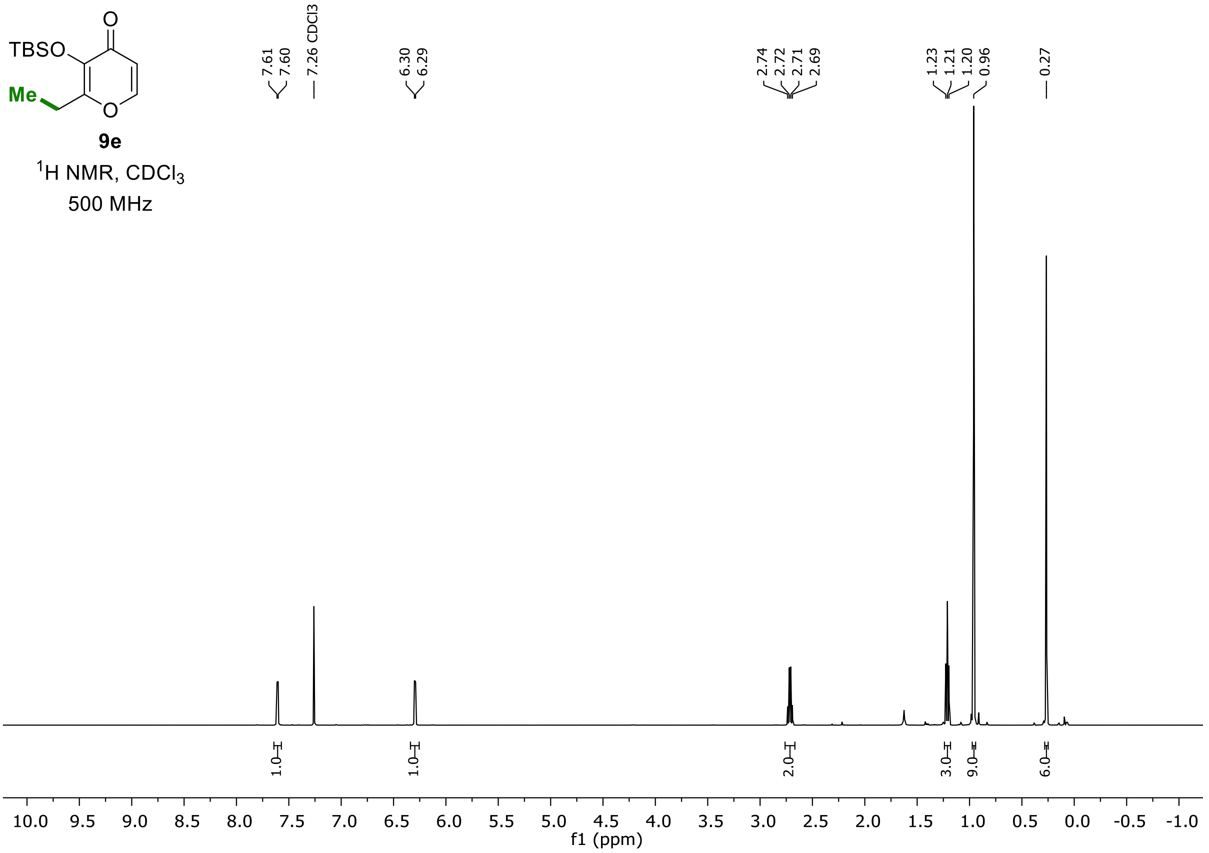
TBSO
O
Me
O
9e
^{1}H NMR, $CDCl_3$
500 MHz
7.61
7.60
7.26 CDCl3
6.30
6.29
2.74
2.72
2.71
2.69
1.23
1.21
1.20
0.96
0.27
1.0
1.0
2.0
3.0
9.0
6.0
f1 (ppm)

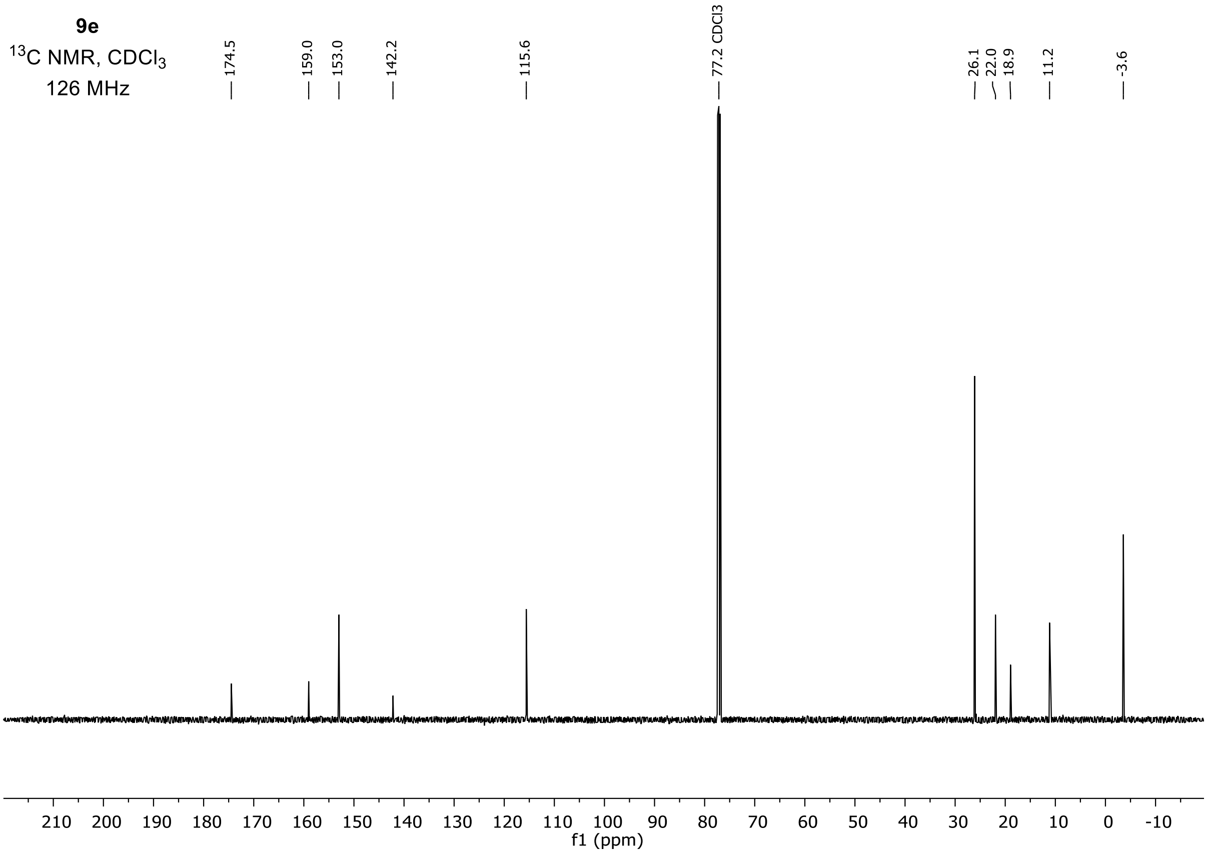
9e
^{13}C NMR, $CDCl_3$
126 MHz
174.5
159.0
153.0
142.2
115.6
77.2 CDCl3
26.1
22.0
18.9
11.2
-3.6
f1 (ppm)

4. References

1. Perez, C.; Daniel, K. B.; Cohen, S. M. *ChemMedChem* **2013**, *8*, 1662–1667. doi:10.1002/cmdc.201300255.

2. Andayi, W. A.; Egan, T. J.; Gut, J.; Rosenthal, P. J.; Chibale, K. *ACS Med. Chem. Lett.* **2013**, *4*, 642–646. doi:10.1021/ml4001084.

3. Foley, D. J.; Craven, P. G. E.; Collins, P. M.; Doveston, R. G.; Aimon, A.; Talon, R.; Churcher, I.; Delft, F. von; Marsden, S. P.; Nelson, A. *Chem. – Eur. J.* **2017**, *23*, 15227–15232. doi:10.1002/chem.201704169.

4. Rumbo, A.; Castedo, L.; Mourino, A.; Mascarenas, J. L. *J. Org. Chem.* **1993**, *58*, 5585–5586. doi:10.1021/jo00073a007.
